# NET1 is a critical regulator of spindle assembly and actin dynamics in mouse oocytes

**DOI:** 10.1186/s12958-023-01177-4

**Published:** 2024-01-02

**Authors:** Shiwei Wang, Xuan Wu, Mengmeng Zhang, Siyu Chang, Yajun Guo, Shuang Song, Shizhen Dai, Keliang Wu, Shenming Zeng

**Affiliations:** State Key Laboratory of Animal Biotech Breeding, National Engineering Laboratory for Animal Breeding, Key Laboratory of Animal Genetics, Breeding and Reproduction of the Ministry of Agriculture, College of Animal Science and Technology, China Agricultural University, Beijing, China

**Keywords:** NET1, RAC1, HACE1, Oocyte, Meiosis

## Abstract

**Background:**

Neuroepithelial transforming gene 1 (NET1) is a RhoA subfamily guanine nucleotide exchange factor that governs a wide array of biological processes. However, its roles in meiotic oocyte remain unclear. We herein demonstrated that the NET1-HACE1-RAC1 pathway mediates meiotic defects in the progression of oocyte maturation.

**Methods:**

NET1 was reduced using a specific small interfering RNA in mouse oocytes. Spindle assembly, chromosomal alignment, the actin cap, and chromosomal spreads were visualized by immunostaining and analyzed under confocal microscopy. We also applied mass spectroscopy, and western blot analysis for this investigation.

**Results:**

Our results revealed that NET1 was localized to the nucleus at the GV stage, and that after GVBD, NET1 was localized to the cytoplasm and predominantly distributed around the chromosomes, commensurate with meiotic progression. NET1 resided in the cytoplasm and significantly accumulated on the spindle at the MI and MII stages. Mouse oocytes depleted of *Net1* exhibited aberrant first polar body extrusion and asymmetric division defects. We also determined that *Net1* depletion resulted in reduced RAC1 protein expression in mouse oocytes, and that NET1 protected RAC1 from degradation by HACE1, and it was essential for actin dynamics and meiotic spindle formation. Importantly, exogenous RAC1 expression in *Net1*-depleted oocytes significantly rescued these defects.

**Conclusions:**

Our results suggest that NET1 exhibits multiple roles in spindle stability and actin dynamics during mouse oocyte meiosis.

**Supplementary Information:**

The online version contains supplementary material available at 10.1186/s12958-023-01177-4.

## Background

Neuroepithelial transforming gene 1 (*Net1*) is a RhoA subfamily-specific guanine nucleotide exchange factor that is involved in diverse biological processes, especially in mitotic progression, cell motility, and most malignant tumors [1, 2]. By activating the Akt-signaling pathway, NET1 promotes hepatocellular carcinoma (HCC) growth and metastasis [3], and NET1 has been demonstrated to be involved in cell-cycle kinetics by stimulating rRNA synthesis and modulating nucleolar structure [4]. Cellular proliferation and chemoresistance are enhanced by *Net1* overexpression, and apoptosis is induced by *Net1* silencing [5, 6]. Additionally, NET1 exerts a key function in cytoskeletal organization, and its upregulation leads to spindle polarity defects [7]. NET1 activity is crucial for driving cytoskeletal rearrangement in tumor cell migration and invasion [8]. However, the function of NET1 in oocyte meiosis remains obscure.

RAC1 is a small G-protein belonging to the Rho subfamily that participates in a multitude of biological processes [9]. It has been shown that RAC1 is critical for the proliferation, differentiation, and steroidogenesis of granulosa cells (GC) in chicken ovarian follicles [10]; and human embryonic implantation requires RAC1, which facilitates stromal cell migration at implantation sites [11]. Inhibition of RAC1 activity with NSC 23,766 in vitro exerts a detrimental effect on porcine oocyte maturation and early embryonic development [12]. However, an in vivo assay revealed that *Rac1* depletion exerted little effect on mouse oocyte meiotic progression and that the resulting mutant mice showed normal fertility [13].

During mammalian oocyte maturation, the quality of oocytes affects the effectiveness of fertilization and embryonic development [14]. For example, to produce a healthy oocyte, spindle assembly and chromosomal alignment must be properly controlled during meiotic progression [15]. Aneuploid eggs can be generated by any error in the oocyte maturation process [16], and several studies have shown that fertilization of aneuploid eggs in mammals leads to pregnancy loss, which—if the fetuses survive to term—results in developmental disabilities [17]. Spindle assembly and asymmetric-division defects are major causes—particularly in women of advanced maternal age—of infertility, miscarriages, or birth defects [18].

A variety of molecules and pathways have been proposed to affect cytoskeletal organization and actin cap formation during oocyte maturation, but the underlying mechanisms have yet to be fully understood. Based on the function of NET1 in somatic cell, we hypothesize that NET1 may play vital role in oocyte maturation. In the present experiment, we explored the NET1 functions during mouse oocyte meiosis by silencing and overexpression of relative genes.

## Materials and methods

If not stated otherwise, all of the chemicals and culture media were obtained from Sigma (St. Louis, MO, USA).

### Mice

During this study, Beijing Sibefu Biotechnology Co., Ltd., provided ICR female mice (6–8 weeks), and these were raised in the specific pathogen free (SPF) Animal Center of the College of Animal Sciences and Technology, China Agricultural University. Experimental animal protocols were conducted in accordance with relevant ethical guidelines and regulations, and were approved by China Agricultural University.

### Antibodies

Mouse monoclonal anti-NET1 antibodies (Cat# 28180-1-AP) was obtained from Santa Cruz Biotechnology (Santa Cruz, CA); rabbit polyclonal anti-RAC1 antibodies (Cat#: 24072-1-AP), mouse polyclonal anti-HACE1 antibodies (Cat# 24104-1-AP), and mouse monoclonal anti-GAPDH antibodies (Cat#: 60004-1-Ig) were purchased from Proteintech (Wuhan, China). Mouse monoclonal α-tubulin-FITC antibody (Cat# F2168) was from Sigma (St. Louis, MO, USA); and anti-c-MYC-antibody (Cat# 2278 S), HRP-conjugated rabbit (Cat #7074S), and mouse (Cat# 7076 S) secondary antibodies were from Cell Signaling Technology (Danvers, MA, USA). We purchased Fluor 555 (Cat# A-11,001) and 488 (Cat# A-21,429) conjugated anti-mouse and anti-rabbit secondary antibodies from Invitrogen (USA).

### Oocyte collection and culture

Mice were superovulated to produce fully grown GV oocytes by injecting them with 10 IU of pregnant mare serum gonadotropin (PMSG) intraperitoneally. After 48 h, we collected GV oocytes from 6–8-week-old mice and placed them in M2 medium (Cat#M7167, Sigma) supplemented with 2.5 µM milrinone to maintain oocyte arrest at the GV stage. The oocytes were subsequently incubated in M16 medium (Cat:MR-016, Sigma) and surrounded by liquid paraffin oil at 37 °C in an atmosphere of 5% CO_2_ in compressed air at high humidity.

### Plasmid construction and mRNA synthesis

We isolated total RNA from 100 denuded GV-stage oocytes using an Arcturus PicoPure RNA Isolation Kit (Applied Biosystems), and cDNA was generated using a QuantiTect Purification Kit (Qiagen, Düsseldorf, Germany). For *Rac1* plasmid construction, PCR products were purified and cloned into the linearized pCS2^+^ vector with six Myc tags using the Uniclone one-step seamless cloning kit (Genesand Biotech Co.,Ltd, Cat# SC612). The plasmid was propagated using Trans1-T1 (Transgen Biotech, Beijing, China) as the host strain and purified with a TIANpure Midi Plasmid Kit (DP107, TIANGEN Biotech, Beijing), followed by sequencing to confirm the open reading frame. For the synthesis of cRNA, the *Rac1* plasmids were linearized with XbaI. Capped cRNAs were achieved using in vitro transcription with SP6 mMESSAGE mMACHINE (Ambion, CA, USA) according to the manufacturer’s instructions. Synthesized RNA was aliquoted and stored at − 80 °C (the related primer sequences can be found in Supporting Information Table S1).

### Quantitative real-time PCR

Total RNA was extracted from 100 oocytes using the RNeasy® Micro Kit (Cat: #157,030,297, Invitrogen, USA), and cDNA synthesis was accomplished using a QuanNova Reverse Transcription Kit (Cat: #205,311, Qiagen, Germany). qPCR was conducted using a Power SYBR Green PCR Master Mix (Applied Biosystems, Life Technologies) with an ABI 7500 Real-Time PCR system (Applied Biosystems). Data were normalized against β-actin, and quantification of fold changes in expression was determined using the comparative CT method as we reported previously (the relevant primers are listed in Table S1).

### Mass spectroscopic analysis

A total of fourteen ovaries were lysed in IP Lysis buffer (Cat: #P0013, Beyotime, Shanghai, China) (20 Mm Tris (pH 7.5), 150 mM NaCl, 1% Triton X-100, mM PMSF, 1×Roche complete mini protease inhibitor cocktail, and 1×Pierce phosphatase-inhibitor cocktail) for 30 min at 4 °C with occasional vortexing. The lysates were cleared and centrifuged at 12,000 × g for 20 min at 4 ℃. Then the supernatant was transferred to a new tube and incubated with Protein A/G Magnetic Beads (MCE, HY-K0202) for overnight. After extensive washing with IP lysis buffer, the protein-beads complexes were transferred to a new 1.5 mL EP tube. Finally, we incubated 500 µL of ovarian lysate with NET1 antibody and bead complexes, and sent the samples to Wayen Biotechnologies, Inc. (Shanghai) for mass spectrometric (MS) analysis (Mass spectrometry data are presented in Supplementary Material 3).

### Microinjection of siRNAs and mRNAs

To explore the functions of *Net1* in mouse oocyte development, specific *Net1*-siRNA and negative control siRNAs were obtained from Shanghai GenePharma CO, Ltd. For the RNAi experiment, siRNAs were diluted to 1 mM with RNase-free dd H_2_O and stored in a − 80 °C refrigerator. The siRNAs were then diluted to 20 μm, and approximately 5–10 pL *Net1*-siRNA solution was microinjected into the oocyte. After siRNA injection the oocytes were arrested at the GV stage in M16 medium containing 2.5 μm milrinone for 20 h to allow time for siRNA-mediated knockdown (KD). For the overexpression experiment, 10 pL of mRNA solution (0.5–1.0 µg/µL) was injected into the cytoplasm of GV oocytes, and the same amount of RNase-free PBS was injected into the controls. After cRNA injection, oocytes were arrested at the GV stage in M16 medium containing 2.5 µM milrinone for 4 h to allow time for overexpression. The oocytes were then transferred to milrinone-free M16 medium for additional experimentation (the siRNA pairs that were used are listed in Supporting Information Table S2).

### Western blot analysis

Oocytes were heated at 100 °C for 10 min in protein lysis buffer (95% Laemmli sample buffer and 5% β-mercaptoethanol) and stored at − 20 °C until needed. Protein lysates were separated on 12% SDS-PAGE gels and then transferred to PVDF membranes. The membranes were blocked in phosphate-buffered saline with Tween 20 (PBST) containing 5% non-fat milk for 1 h at room temperature. Thereafter, membranes were incubated with primary antibodies against NET1 (1:500), RAC1 (1:2000), and GAPDH (1:5000) overnight at 4 °C. After washing three times with Tris Buffered Saline with Tween 20 (TBST) for 10 min each, the membranes were incubated with with horseradish peroxidase-conjugated secondary antibodies (1:5000) at room temperature for 1 h. The membranes were washed three times with Tris-buffered saline with Tween 20 (TBST). The specific proteins were visualized using high-quality ECL Western blotting system (Epizyme, Shanghai, China). For quantitation of Western blot band intensity, densitometric analysis was performed using Image J software and normalized to either the levels of GAPDH.

### Immunofluorescence analysis

Immunofluorescence was performed as described previously [19]. Briefly, oocytes were fixed in phosphate-buffered saline (PBS) containing 4% paraformaldehyde for 30 min at room temperature, permeabilized in PBS containing 0.5% Triton X-100 for 20 min, and blocked in PBS containing 1% bovine serum albumin for 1 h at room temperature. Samples were subjected to indirect immunofluorescence staining by incubation overnight at 4 °C with primary antibodies in blocking solution (NET1, 1:100). After three washes with PBS containing 0.1% and 0.01% Triton X-100, oocytes were incubated with Alexa Fluor 488-conjugated goat anti-rabbit/mouse IgG (1:300) at room temperature for 1 h. To visualize the spindle, oocytes were probed with FITC-conjugated tubulin antibody (1:300). Finally, oocytes were counterstained PI for 10 min. Oocytes were mounted on glass slides and observed using a confocal laser-scanning microscope (Zeiss LSM 710 META). For F-actin staining, the samples were fixed with 3.7% paraformaldehyde for 5 min and then blocked with 1% BSA for 1 h. After incubation with FITC-conjugated phalloidin for 1 h, the DNA was counterstained with PI or Hoechst 33,342 for 10 min. Fluorescence intensities were analyzed using ZEN lite 2012 or ImageJ software (NIH, USA).

### Chromosomal spreads

Chromosomal spreads were created as described previously [16]. Briefly, the zonae pellucidae of oocytes were removed with Tyrode’s acid solution (Cat# T1788-100ML; Sigma, St. Louis, USA) and transferred to M2 medium for recovery. Oocytes were then fixed in 1% paraformaldehyde containing 0.15% Triton X-100. After drying at room temperature, slides containing oocytes were blocked with 1% BSA in PBS for 1 h, nuclear DNA was stained using Hoechst 33,342, and the samples were visualized using an inverted confocal microscope (LSM 710; Carl Zeiss, Germany) affixed with a × 60 objective.

### Statistical analysis

All of the experiments were repeated at least three times. Unpaired, two- tailed Student’s t test were applied to assess differences between two groups. Multiple comparisons between more than two groups were analyzed a one-way ANOVA and Tukey’s multiple-comparison test using GraphPad Prism 5 software. Data are expressed as mean ± SD. *P* < 0.05 was considered to be statistically significant.

## Results

### Subcellular distributions and expression levels of NET1 during oocyte meiosis

**Fig. 1:**
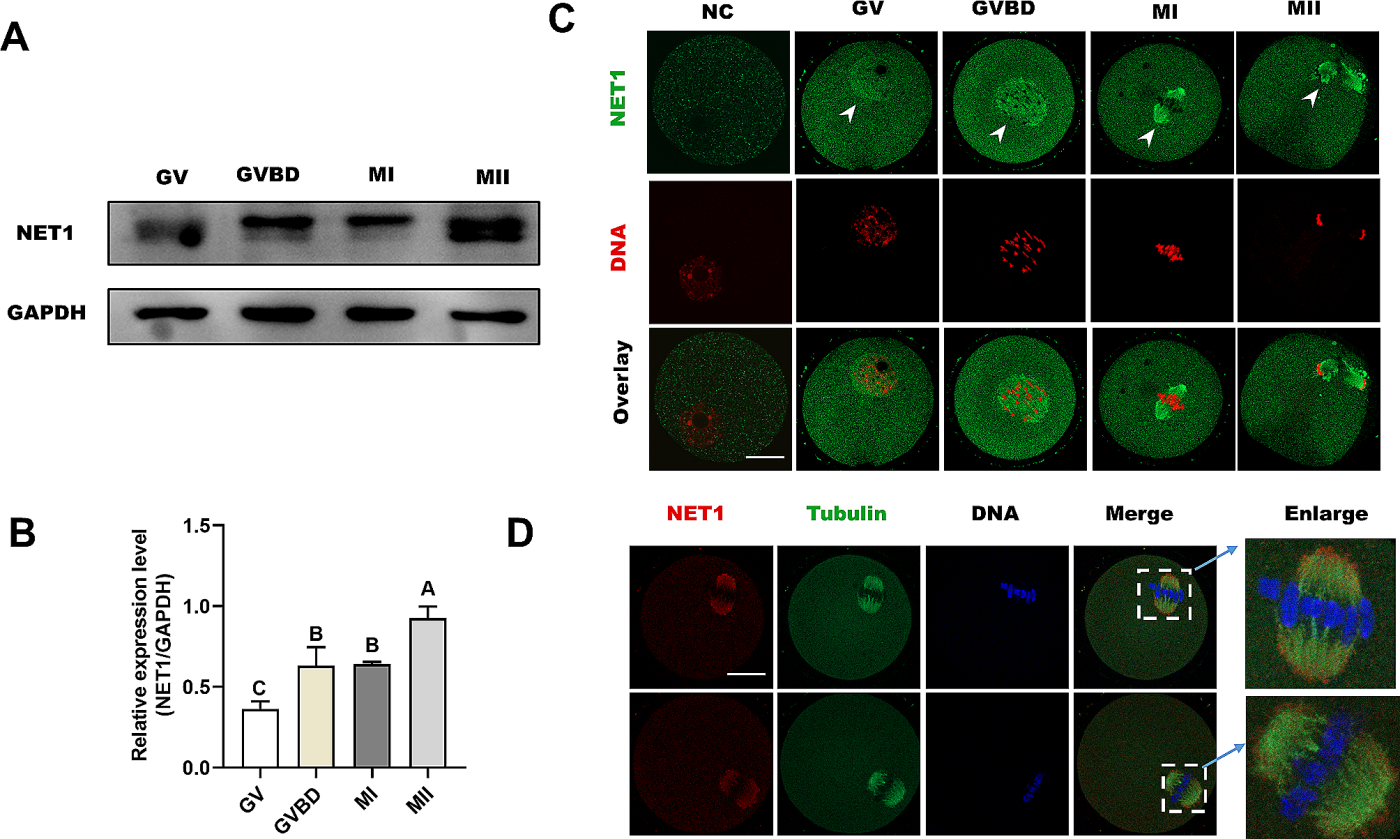
Subcellular localization and expression of NET1 during oocyte maturation. (**A**) Expression of NET1 during meiotic maturation at GV, GVBD, MI, and MII stages (the molecular mass of NET1 is 64 kDa). Proteins from 100 oocytes were loaded for each sample. (**B**) Relative intensity results for NET1 protein expression in GV, GVBD, MI, and MII oocytes. (**C**) Confocal microscopy showing immunostaining for NET1 (green) and DNA (red) in oocytes at GV, GVBD, MI, and MII stages (scale bar = 20 μm). (**D**) Double-labeling of metaphase oocytes with NET1 (red) and α-tubulin antibody (green), and counterstaining of DNA with Hoechst 33,342 (blue) (scale bars, 20 μm)

Mouse oocytes were cultured for 0 h, 3 h, 8 h, and 14 h in M16 medium corresponding to the timepoints when most oocytes reached the GV, GVBD, MI, and MII stages, respectively. To explore the roles of NET1 during mouse meiotic progression, we observed its expression levels and localization patterns at different developmental stages. Western blot analysis suggested that the expression levels of NET1 were gradually increased from the GV stage to the MII stage (Fig. 1A and B). Immunofluorescence and confocal imaging showed that NET1 was primarily distributed to the nucleus at the GV stage (Fig. 1C, arrows), and that after GVBD NET1 was localized to the cytoplasm and predominantly distributed around the chromosomes. However, commensurate with meiotic progression, NET1 resided in the cytoplasm and accumulated significantly on the spindle at the MI and MII stages. In order to confirm the spatial relationship between the spindle and NET1, double staining was performed. As shown in Fig. 1D, there is indeed cololization of NET1 with the meiotic spindle in mouse oocytes. Collectively, the dynamic distribution and expression patterns imply that NET1 plays a unique role in regulating oocyte meiotic maturation.

### NET1 assures meiotic progression in mouse oocytes

**Fig. 2:**
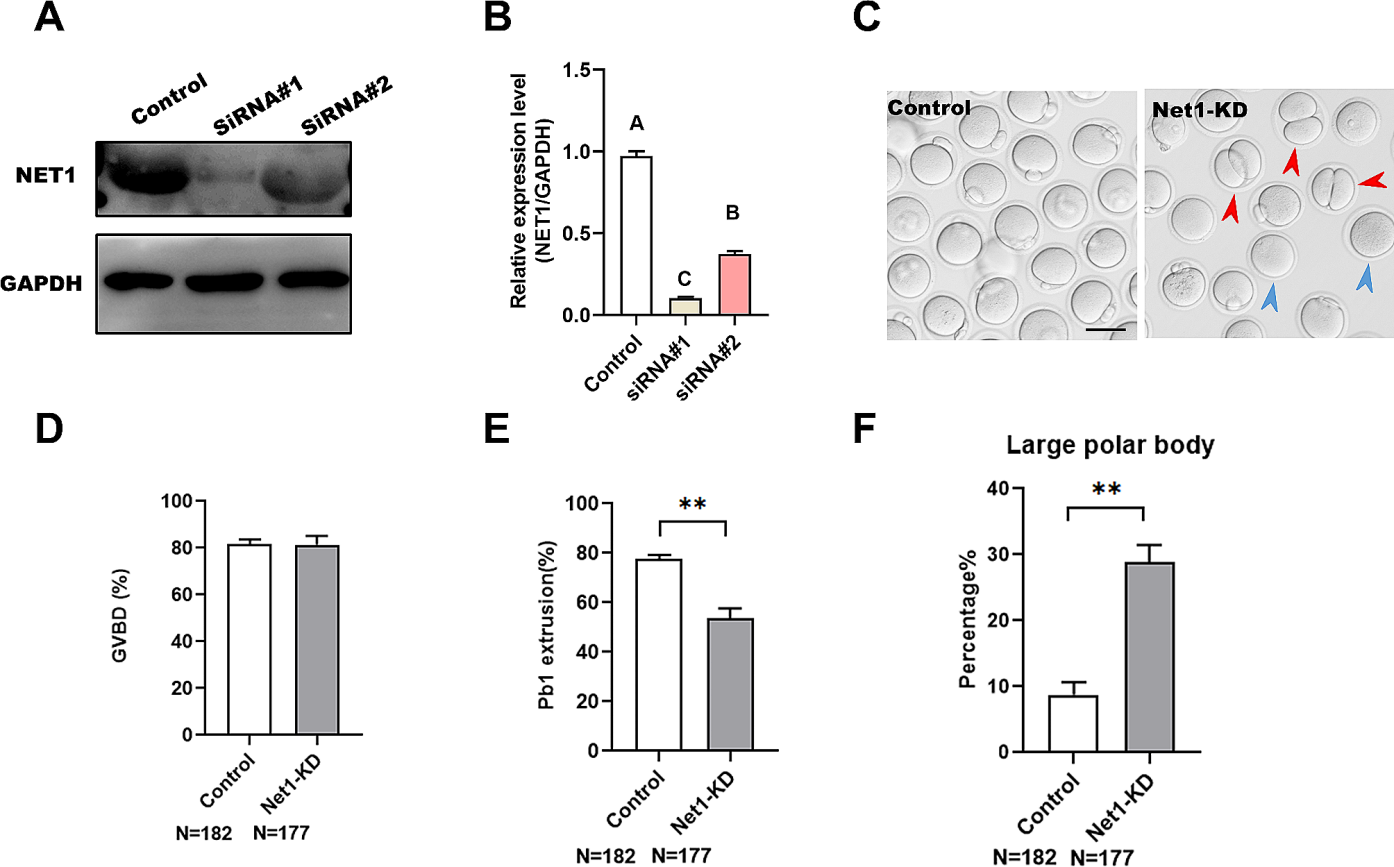
NET1 guarantees meiotic progression in mouse oocytes. (**A**) KD of endogenous NET1 protein after *Net1*-siRNA injection was confirmed by western blot analysis. (**B**) Relative intensity results for the NET1 protein expression in control and *Net1*-KD oocytes. (**C**) Representative images of oocytes from control and *Net1*-KD oocytes. Red arrowheads denote oocytes with apparent symmetrical division and blue arrowheads indicate oocytes that fail to extrude polar bodies (scale bar, 80 μm). (**D**, **E**). Quantitative analysis of GVBD and Pb1 extrusion rates between control and *Net1*-KD oocytes (GVBD, 81.7 ± 1.64% vs. 81.4 ± 3.14% ; Pb1, 77.5 ± 1.41% vs. 31.2 ± 2.48%). (**F**). The percentage of oocytes with large polar bodies after *Net1*-siRNA injection (8.7 ± 1.55% vs. 28.9 ± 2.5%). The Figure depicts the mean percentage ± SD of the results obtained from three independent experiments (*significantly different at *P* < 0.05, **significantly different at *P* < 0.01)

The specific localization and expression patterns of *Net1* in the meiotic process prompted us to investigate whether its knockdown affected the meiotic apparatus in oocytes. To investigate this hypothesis, we microinjected specifically designed *Net1*-siRNAs into fully grown GV oocytes, with a negative siRNA injected as a control. After injection, oocytes were arrested at the GV stage for 16 h in M16 medium containing 2.5 μm milrinone to allow the degradation of endogenous *Net1* mRNA. Based on western blot results, we found that siRNA#1 induced the most substantial reduction in NET1 protein in oocytes (Fig. 2A), and we therefore adopted siRNA#1 in subsequent KD experiments. Of note, we found that the siRNA-injected oocytes displayed wide variation in NET1 staining (Fig. S1). After 3 h of culture, both control and *Net1*-KD oocytes resumed meiosis (Fig. 2C). However, after 14 h of culture, only 31.2% of the *Net1*-KD oocytes extruded Pb1, which was significantly less than the rate for controls (Fig. 2B). We also observed a large proportion of oocytes that extruded a polar body that was larger than normal (the polar body diameter was greater than 1/3 of the oocyte’s diameter) in the *Net1-*depleted oocytes compared with the control oocytes at the MII stage (Fig. 2B and E, red arrows). The large polar body and symmetric division of oocytes constituted the chief phenotype related to failure of spindle migration during meiosis I [20]. Collectively, these results suggested that *Net1*-KD impaired meiotic maturation and induced asymmetric division in mouse oocytes.

### NET1 is essential for spindle assembly, K-MT attachment, and cytoskeletal organization in mouse oocytes

**Fig. 3:**
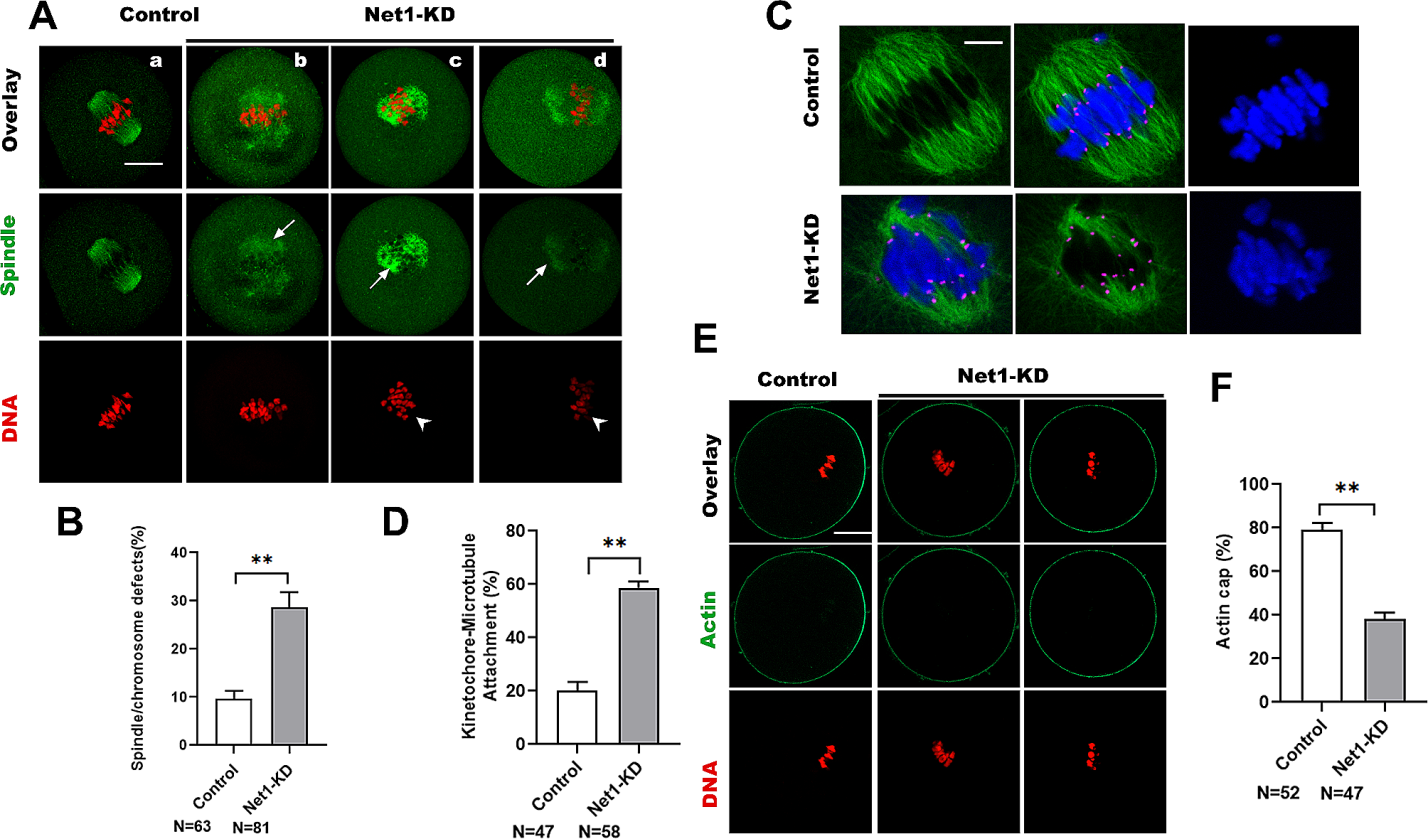
NET1 is essential for spindle assembly, K-MT attachments, and cytoskeletal organization in mouse oocytes. (**A**) Control and *Net1*-KD oocytes were stained with α-tubulin antibody to visualize the spindle (green) and counterstained with PI to observe chromosomes (red). (a) Control oocytes show the characteristic barrel-shaped spindles (arrows) and well-aligned chromosomes. (b-d) Three examples illustrating the disorganized spindles (arrows) and misaligned chromosomes (arrowheads) that were frequently observed in *Net1*-KD oocytes (scale bar, 20 μm). (**B**). Quantification of control and *Net1*-KD oocytes with spindle/chromosomal defects (9.6 ± 1.39% vs. 28.9± 2.58%). (**C**). Control and *Net1*-KD metaphase oocytes were labeled with CREST antibody for kinetochores (purple), anti-tubulin antibody for microtubules (green), and Hoechst 33,342 for chromosomes (blue). Representative confocal images are shown. (**D**). Quantitative analysis of K-MT attachments in control and *Net1*-KD oocytes (20.1 ± 2.70% vs. 58.7 ± 1.99%). Kinetochores in regions where fibers were not easily visualized were not included in the analysis. (**E**) Metaphase I oocytes were labeled with phalloidin to visualize actin (green) and counterstained with propidium iodide for chromosomes (red). (**F**) Quantification of control and *Net1*-KD oocytes with formation of a normal actin cap (79.2 ± 2.55% vs. 38.2 ± 2.45%). The Figure depicts the mean percentage ± SD of the results obtained from three independent experiments (*significantly different at *P* < 0.05, **significantly different at *P* < 0.01)

The unique distribution of NET1 on the spindle and its effects on oocyte maturational progression precipitated our investigation of whether *Net1* depletion in oocytes influenced assembly of the meiotic apparatus. Control and *Net1*-KD oocytes were immunolabeled with anti-a-tubulin antibody to visualize the spindle and counterstained with PI to visualize chromosomes. *Net1* KD elevated the percentage of spindle defects and chromosomal misalignment in metaphase oocytes (Fig. 3A and B), displaying defective spindles (arrows) and scattered chromosomes (arrowheads). These phenotypes contrasted sharply with control oocytes that exhibited a typical barrel-shaped spindle and well-aligned chromosomes at the cellular equator. In order for chromosomes to be properly aligned and segregated, kinetochores must be attached to microtubules emanating from the opposite spindle pole [16]; however, we noted that *Net1*-KD oocytes exhibited spindle/chromosome disorganization and hypothesized that NET1 may be required for correct kinetochore-microtubule (K-MT) assembly. CREST was used to detect kinetochores, anti-α-tubulin antibody was used to visualize spindles, and Hoechst 33,342 was used to visualize chromosomes in MI oocytes (Fig. 3C). We ascertained that the majority of normal oocytes manifested the typical amphitelic K-MT attachments, but that *Net1*-KD oocytes reflected a high frequency of K-MT misattachments (Fig. 3D). In addition, actin polymerization participates in spindle migration, actin cap formation, and polar body extrusion [21]. Therefore, to determine whether *Net1*-KD affected actin polymerization, actin was labeled with phalloidin (Fig. 3E); and as shown in Fig. 3F, actin caps were visible in approximately 79% and 38% of control and *Net1*-KD oocytes, respectively. We therefore speculated that *Net1* KD impaired cytoskeletal organization due to errors in K-MT attachments.

### NET1 deficiency activates the SAC and increases the likelihood of aneuploidy

**Fig. 4:**
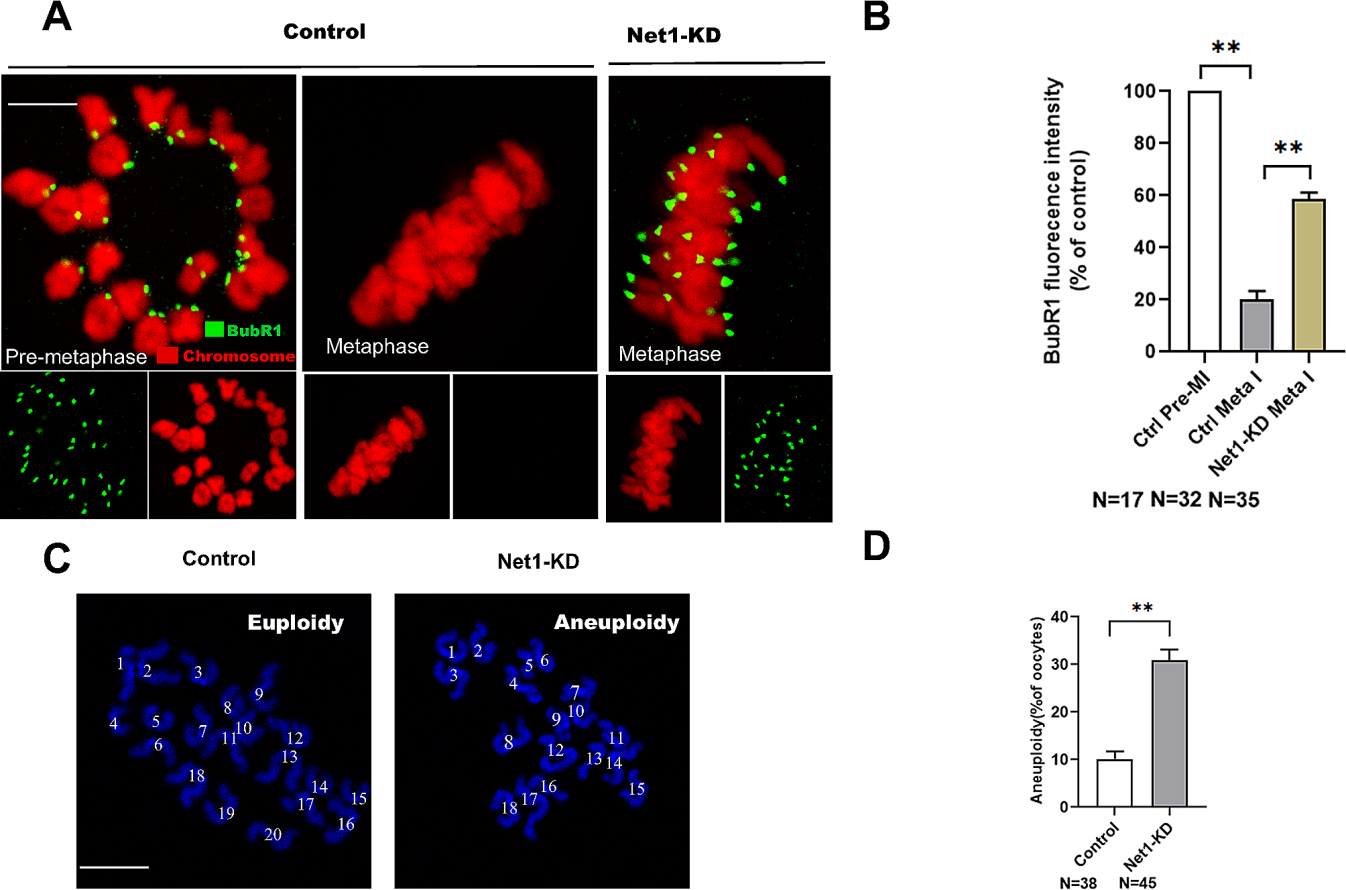
Reduced Net1 activates the SAC and increases the incidence of aneuploidy. (**A**) Control and *Net1*-KD oocytes were immunolabeled with anti-BubR1 antibody (green) and counterstained with PI to examine chromosomes (red). Representative confocal images of pre-MI and MI oocytes are shown. Arrowheads indicate scattered chromosomes in *Net1*-KD oocytes (scale bar, 2.5 μm). (**B**) Quantification of BubR1 fluorescence intensity in control and *Net1*-KD oocytes (19.3 ± 2.14% vs. 71.5 ± 1.48%). (**C**) Chromosomal spreads of control and *Net1*-KD MII oocytes (chromosomes were stained with Hoechst 33,342 [blue]). Representative confocal images show euploid control oocytes and aneuploid *Net1*-KD oocytes. Arrows indicate the premature separation of sister chromatids (scale bar, 2.5 μm). (**D**) Quantification of aneuploidy in control and *Net1*-KD oocytes (10.1 ± 1.33% vs. 30.9 ± 1.85%). Graph shows the mean ± SD of the results obtained from three independent experiments (*significantly different at *P* < 0.05, **significantly different at *P* < 0.01)

In order to ensure accurate chromosomal segregation, the spindle assembly checkpoint (SAC) monitors kinetochore-microtubules interactions [22]; and SAC signals are triggered by kinetochore-microtubule attachment disturbances [23]. *Net1*-KD oocytes exhibited impaired K-MT attachments and meiosis I arrest, suggesting the SAC may be provoked when *Net1* is depleted. For the purpose of testing this hypothesis, the SAC activity was evaluated in *Net1*-KD and normal oocytes that were immunolabeled with BubR1, an integral component of the checkpoint complex. As kinetochores were typically attached to microtubules and BubR1 appeared continuously on unattached kinetochores during pre-metaphase, but disappeared completely once kinetochores were attached (Fig. 4A and B). The SAC was activated in *Net1*-KD oocytes arrested at meiosis, since the BubR1 signal at the kinetochores was dramatically elevated. Overall, these erroneous K-MT attachments were likely to be the primary cause of chromosomal misalignment and activation of SAC in *Net1*-KD oocytes.

SAC normally delays anaphase onset until all chromosomes are aligned at the equatorial plane and attached to spindles [24], and the major driving force behind aneuploidy is deregulated SAC [25]. The high frequency of spindle/chromosomal defects in *Net1*-KD oocytes led us to assess whether aneuploidy was also associated with this defect. For this experiment, MI oocytes were processed to spread chromosomes, with representative images of euploidy and aneuploidy shown in Fig. 4C. *Net1* KD resulted in a three-fold increase in aneuploid eggs compared to controls (Fig. 4D). According to these results, *Net1* knockdown disrupted spindle/chromosome organization and induced SAC during meiosis, leading to aneuploid eggs.

### NET1 is associated with RAC1

**Fig. 5:**
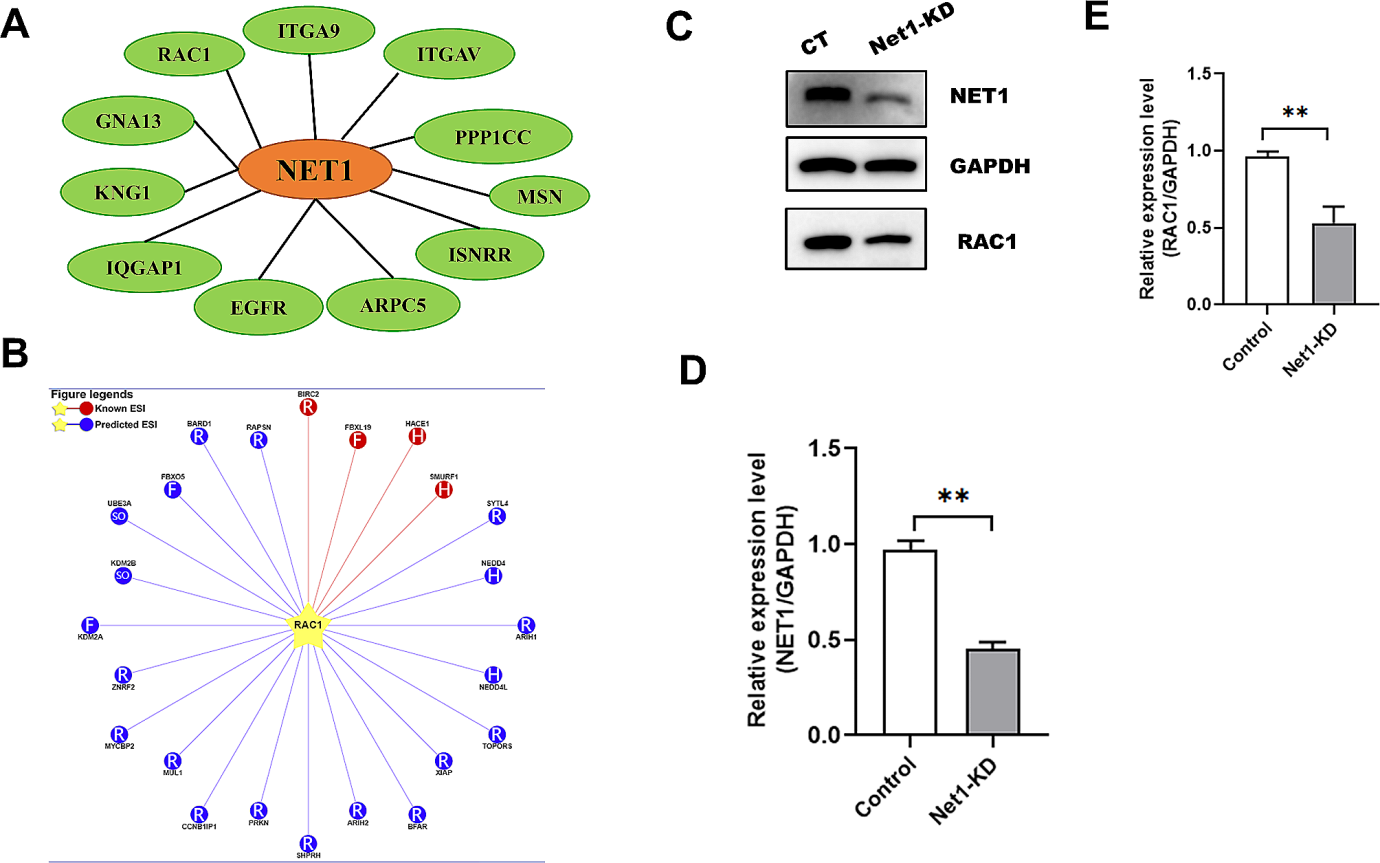
NET1 is associated with RAC1. (**A**) Screening of actin-related proteins correlated with NET1 by mass spectrometric analysis. (**B**) Prediction of E3 ubiquitin ligase targeting RAC1 degradation. (**C**) RAC1 protein levels in control and *Net1-*KD oocytes. (**D**) Relative-intensity results for NET1 protein expression in the control and *Net1*-KD oocytes. (**E**) Relative-intensity results for RAC1 protein expression in the control and *Net1*-KD oocytes

To explore the regulatory mechanism underlying NET1’s actions on oocyte maturation, we conducted mass spectrometric analysis and uncovered several actin-related proteins that were associated with NET1—including RAC1, GNA13, ARPC5, and IQGAP1 (Fig. 5A, Table S3). Considering the importance of RAC1 in the regulation of oocyte polarity [26], we noted that *Net1* knockdown caused disruption of asymmetric division and abnormal actin cap formation in mouse oocytes. When RAC1 protein expression was then assessed by western blotting in *Net1*-KD oocytes, we found it to be significantly reduced (Fig. 5C). *Net1*-KD oocytes also displayed phenotypes highly similar to RAC1-inhibited oocytes [12], and we hypothesized that reduced RAC1 was responsible for the meiotic defects associated with *Net1* depletion. Overall, our findings suggested that reciprocal interactions between NET1 and RAC1 were crucial for spindle assembly and asymmetric division during meiosis.

### NET1 protects RAC1 from HACE1-mediated degradation

**Fig. 6:**
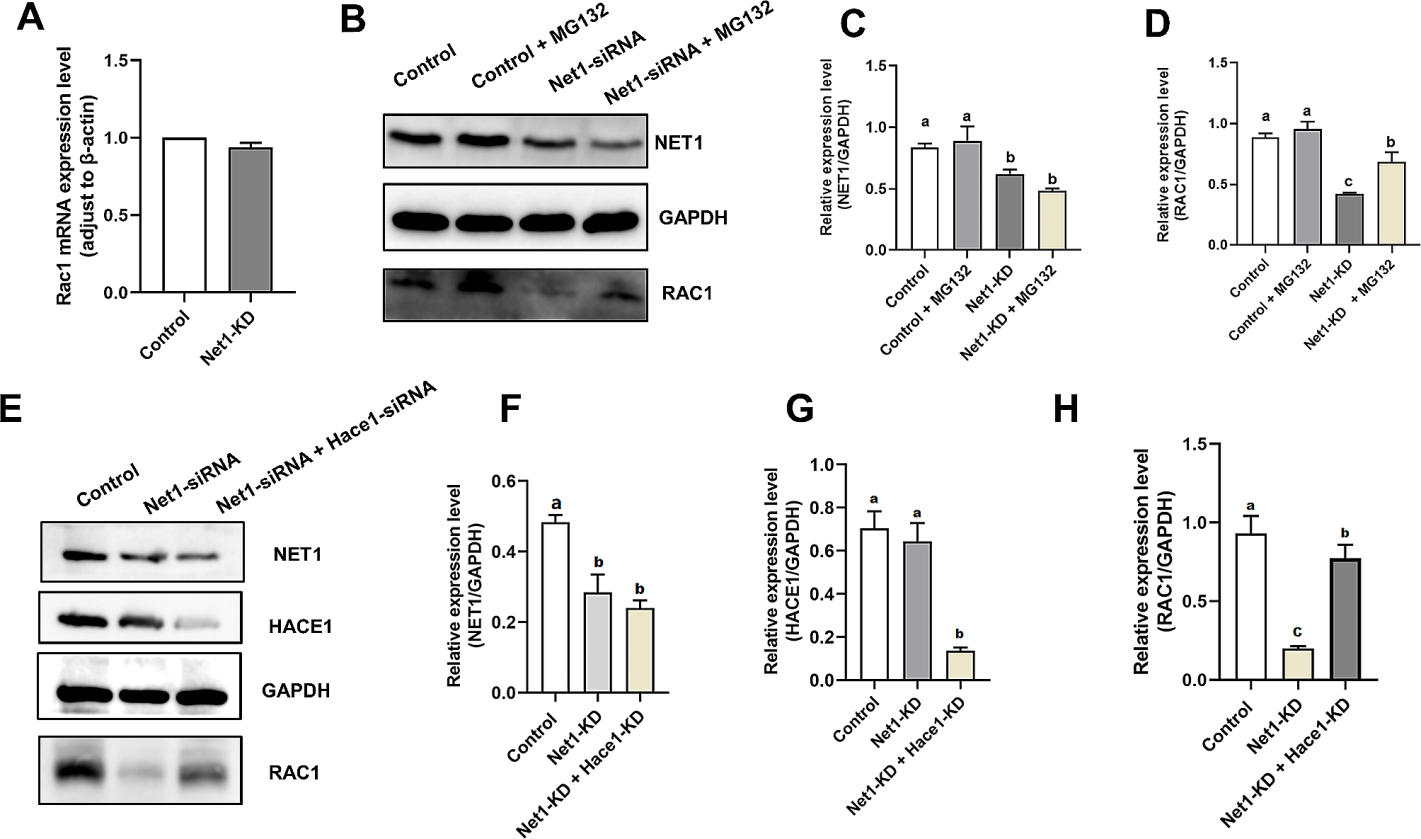
NET1 protects RAC1 from HACE1-mediated degradation. (**A**) *Rac1* mRNA expression levels in Net1-KD and control oocytes (n = 50 per group). (**B**) RAC1 protein levels in the control, *Net1*-KD, and *Net1*-KD + MG132 oocytes. The immunoblots were probed with NET1, RAC1, and GAPDH antibodies. (**C**) Relative- intensity results for NET1 protein expression in the control, *Net1*-KD, and *Net1*-KD + MG132 oocytes. (**D**) Relative-intensity results for RAC1 protein expression in the control, control + MG132, *Net1*-KD, and *Net1*-KD + MG132 oocytes. (**E**) RAC1 protein levels in the control, *Net1*-KD, *Net1*-KD + *Hace1*-KD oocytes. The immunoblots were probed with NET1, RAC1, and GAPDH antibodies

The E3 ubiquitin ligase HECT domain and ankyrin repeat containing E3 ubiquitin protein ligase 1 (HACE1) is a potent tumor suppressor that controls cellular proliferation and ubiquitylates the small GTPase RAC1 to target it for proteasomal degradation [27]. There were no significant changes in *Rac1* mRNA levels after *Net1*-KD (Fig. 6A), but protein levels were greatly reduced (Fig. 5C). We therefore hypothesized that *Net1* depletion activated ubiquitin-proteasomes and mediated RAC1 degradation. We substantiated our hypothesis by inhibiting ubiquitin proteasomal activity in *Net1*-depleted oocytes with MG132 (5 µM, Cat# M8619; Sigma, St. Louis, USA), a proteasomal inhibitor, and ascertained that RAC1 protein was partially recovered from *Net1*-depleted oocytes treated with MG132 (Fig. 6B and D). In order to further determine whether HACE1 was involved in the degradation of RAC1, GV oocytes were microinjected with *Net1*-siRNA or with PBS as a control; and *Net1-*KD GV oocytes were microinjected with HACE1 siRNA. We thereby proved that RAC1 protein was partially recovered in the *Net1*-siRNA + *Hace1*-siRNA-injected oocytes (Fig. 6E and H), suggesting that reduced RAC1 in response to *Net1*-KD was due to increased HACE1 activity.

### Ectopic RAC1 expression in Net1-depleted oocytes rescued meiotic defects

**Fig. 7:**
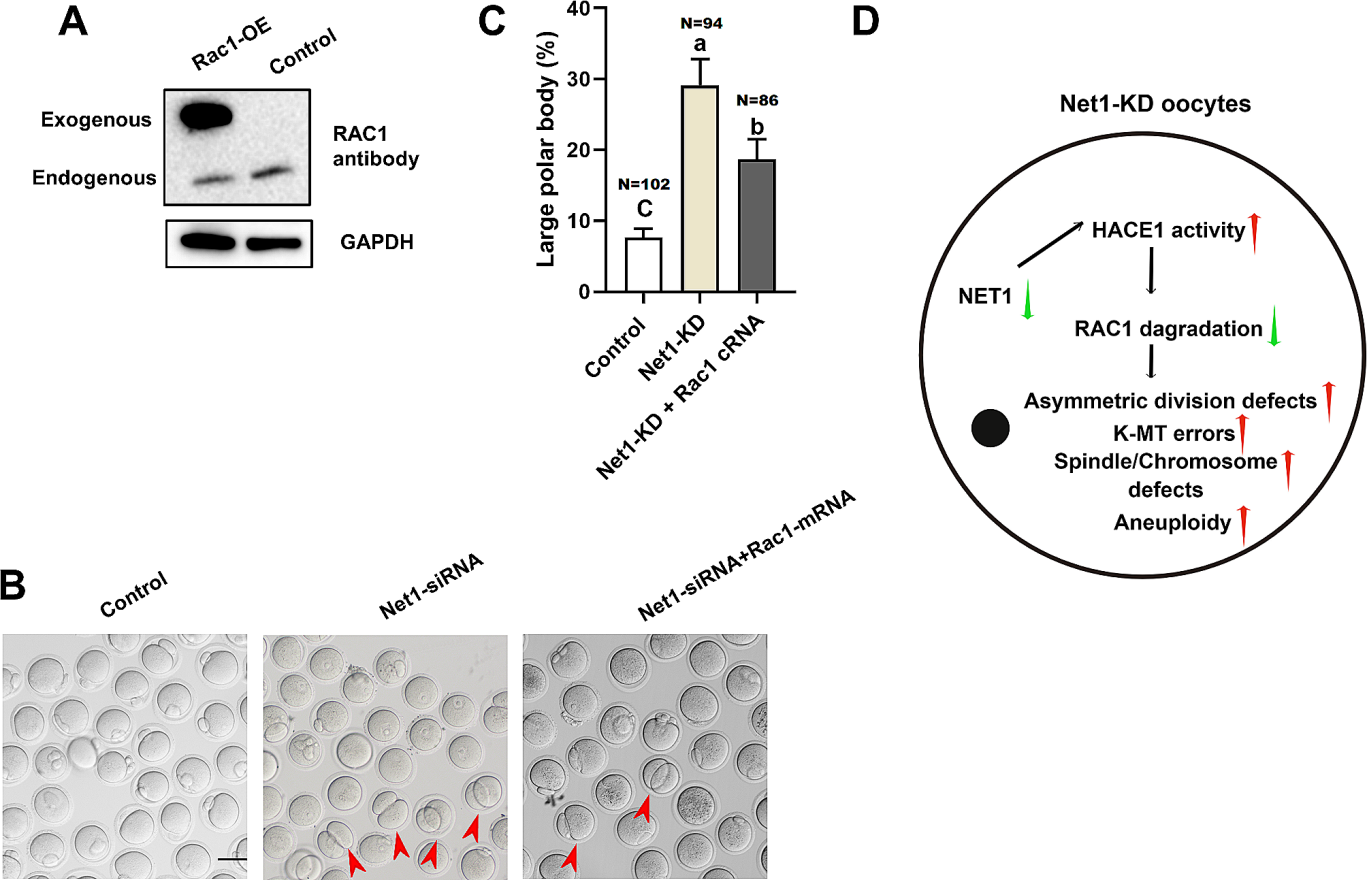
Ectopic RAC1 expression in *Net1*-depleted oocytes rescued meiotic defects. (**A**) Western blotting analysis showed that exogenous Myc-Rac1 protein was efficiently overexpressed by blotting with anti-RAC1 antibody. Bands of endogenous and exogenous RAC1 protein are indicated. (**B**) Representative images of control, *Net1*-KD and *Net1*-KD + *Rac1*-OE oocytes. Red arrowheads denote oocytes with apparent symmetrical division and blue arrowheads indicate oocytes that fail to extrude polar bodies (scale bar, 80 μm). (**C**) The percentage of oocytes with large polar bodies in the control, *Net1*-siRNA and *Net1*-siRNA + *Rac1* mRNA injection (7.6 ± 1.02% vs. 29.1 ± 3.05% vs. 18.7 ± 2.33%). (**D**) Proposed model for the role of NET1 in spindle assembly and actin dynamics in mouse oocytes. When the NET1 level was reduced in oocytes, increased HACE1 activity promoted RAC1 degradation and precipitated meiotic abnormalities in mouse oocytes

We therefore asked whether the reduced RAC1, at least in part, mediates the effects of NET1 on oocyte maturation. Towards this goal, we ectopic expression of *Rac1* in *Net1*-KD oocytes. Immunoblotting verified that exogenous RAC1 protein was efficiently overexpressed (Fig. 7A). To our surprise, ectopic expression of *Rac1* dramatically alleviated the asymmetric defects (Fig. 7B and C) in *Net1-*KD oocytes. Collectively, our data showed that *Net1*-KD-induced meiotic defects were partially due to HACE1-mediated RAC1 protein degradation (Fig. 7D).

## Discussion

NET1 is a guanine nucleotide exchange factor specific for the small GTPase Rho and participates in diverse biological processes [28], including cytoskeletal dynamics [7], cancer [29] and DNA damage [30]. In the present study, we observed a dynamic localization of NET1 during meiotic progression in mouse oocytes. We demonstrated that NET1 predominantly accumulated on the spindle poles in concert with meiotic resumption (Fig. 1B), which was congruent with previous findings in somatic cells [31]. Based on its unique localization and expression dynamics in oocytes, NET1 is hypothesized to portray unique or additional roles in meiosis.

In support of this speculation, we further noted that *Net1* depletion in oocytes disrupted spindle assembly and chromosomal alignment, as well as K-MT interactions (Fig. 3). As reliable chromosomal separation is ensured by the bi-oriented interaction of chromosomes with the spindle through the end-on attachment of microtubules to kinetochores [32], it is conceivable that the high percentage of spindle/chromosomal defects in *Net1*-depleted oocytes is a consequence of K-MT misattachments. If these attachment errors are not corrected prior to anaphase in normal oocyte maturation, they may cause chromosome-separation defects [16]; and consistent with this concept, the frequency of aneuploidy was significantly increased in *Net1*-depleted oocytes relative to controls (Fig. 4C). We then proposed that compromised K-MT stability in *Net1*-KD oocytes might, at least in part, have contributed to the meiotic defects we observed and the generation of the aneuploid eggs we noted in our experiments.

Before fertilization, mammalian oocytes undergo a highly asymmetric division to retain most maternal stores during meiosis by extruding a small polar body [33]. *Net1* KD reduced cellular proliferation and alternations in cell cycle, and inhibited actin fiber formation [34]. Our results also showed that abnormal oocyte meiotic progression was accompanied by a higher proportion of larger polar bodies after *Net1* depletion (Fig. 2), indicating that NET1 was a critical regulator of oocyte maturation, particularly in asymmetric division. We therefore subsequently implemented mass spectrometry to search for NET1-interacting proteins, and our mass spectrometric data also revealed some cytoskeletal proteins—including RAC1, GNA13, ARPC5, and IQGAP1 (Fig. 5A). RAC1 is required for human endometrial stromal cell migration, and *Rac1* KD in these cells inhibited human embryonic trophoblast invasion into stromal cell monolayers [11]. Silencing RAC1 activity suppressed cellular proliferation and promoted apoptosis of ovarian granulosa cells [35]. RAC1 is also critical to hen ovarian follicle growth and development [10]. Our further studies revealed that RAC1 levels were significantly attenuated in *Net1*-depleted oocytes (Fig. 5C). Inhibition of RAC1 during oocyte maturation caused a permanent block at prometaphase I and spindle elongation [26], and RAC1 activity was demonstrated to be essential for porcine oocyte maturation and early embryonic development [12]. In addition, RAC1 is able regulate a large variety of disparate functions, including the organization of the actin cytoskeleton, cell-cycle progression, cell survival, and apoptosis [35–37]. We were therefore intrigued in determining how NET1 mediated the degradation of RAC1.

HACE1 is a recognized E3 ubiquitin ligase that degrades RAC1 [38]. *Hace1-*deficient mice were more susceptible to spontaneous and induced tumors [39], and lung tumorigenesis in *Hace1*-deficient mice was associated with elevated RAC1 activity and enhanced ROS levels [40]. We predicted the E3 ubiquitin ligases capable of degrading RAC1 via the website http://ubibrowser.ncpsb.org/, and HACE1 was among them. Given the changes in *Rac1* mRNA and protein levels in *Net1*-KD and control oocytes (Figs. 5C and 6A), we speculated that *Net1* depletion activated HACE1 activity, thereby leading to the degradation of RAC1. Our mass spectroscopy data confirmed a interaction between NET1 and RAC1 (Fig. 5A), and our western bloy verifying that NET1 was required to preserve RAC1 protein levels in mouse oocytes (Fig. 5C). We were subsequently interested in determining RAC1 protein levels in *Net1*-KD oocytes, and observed that RAC1 protein expression increased in these oocytes and that the proteasomal inhibitor MG132 phenocopied their RAC1 protein levels—implying that NET1-mediated degradation of RAC1 occurred via the ubiquitin-proteasome pathway. RAC1 degradation was eliminated when the siRNA we applied reduced the levels of *Hace1*, and ectopic expression of *Rac1* dramatically alleviated the asymmetric defects (Fig. 7B and C) in *Net1-*KD oocytes, suggesting that HACE1 promoted RAC1 degradation.

In summary, our results revealed that NET1 is an actin cytoskeletal regulator required for meiotic maturation in mouse oocytes. We observed that defects in spindle assembly and asymmetric cell division of *Net1-*KD oocytes was partially due to reduced RAC1, and that NET1 protected RAC1 from HACE1- mediated degradation.

## Conclusions

Our data collectively indicated that NET1 is essential in spindle assembly and actin dynamics during mouse oocyte maturation. NET1 KD activated HACE1 activity through ubiquitin proteasome-mediated RAC1 degradation, which is one of the reasons for abnormal spindle formation and asymmetric division defects (Fig. 7).

### Electronic supplementary material

Below is the link to the electronic supplementary material.

Supplementary Material 1

Supplementary Material 2

Supplementary Material 3

## Data Availability

The data used to support the findings of this study are available from the authors upon reasonable request.

## Abbreviations

NET1: Neuroepithelial transforming gene 1
RAC1: Rac family small GTPase 1
GV: Germinal vesicle
GVBD: Germinal vesicle breakdown
MI: Metaphase I
MII: Metaphase II, KD, knockdown
KD: Knockdown
