# Supplementary material for: NET1 is a critical regulator of spindle assembly and actin dynamics in mouse oocytes
Source: Reprod Biol Endocrinol. 2024 Jan 2;22:5. doi: 10.1186/s12958-023-01177-4 (PMC10759572; doi:10.1186/s12958-023-01177-4)
Supplement: Supplementary file 2 — Supplementary Material 2 [file 12958_2023_1177_MOESM2_ESM.docx]

**Supplemental Figures and Tables for**

**NET1 is a critical regulator for spindle assembly and actin dynamics in mouse oocytes**

Shiwei Wang, Xuan Wu, Mengmeng Zhang, Siyu Chang, Yajun Guo, Shuang Song, Shizhen Dai, Keliang Wu, Shenming Zeng*

State Key Laboratory of Animal Biotech Breeding, National Engineering Laboratory for Animal Breeding, Key Laboratory of Animal Genetics, Breeding and Reproduction of the Ministry of Agriculture, College of Animal Science and Technology, China Agricultural University, Beijing, China

*Corresponding author: [zengsm@cau.edu.cn](mailto:zengsm@cau.edu.cn)

Phone: 18310106460

**This file includes:**

Tables. S1 and S2

Fig. S1

Supplementary Table 1. Primer sequences used for cDNA amplification and qPCR-directed mutagenesis.

| Gene | Primer sequence |
| --- | --- |
| *Rac1*-WT-F | 5’- TCATTTCTGAAGAGGACTTGAATTCATGCAGGCCATCAAGTGTGT -3’ |
| *Rac1*-WT-R | 5’- TAATACGACTCACTATAGTTCTAGATTACAACAGCAGGCATTTTC -3’ |
| *Rac1* -F | 5’- CCTATCATCCTCGTGGGGAC-3’ |
| *Rac1*-R | 5’- CTTGAGTCCTCGCTGTGTGA-3’ |
| β-*actin*-F | 5’-GGCTGTATTCCCCTCCATCG-3’ |
| β-*actin*-R | 5’-CCAGTTGGTAACAATGCCATGT-3’ |

Supplementary Table 2. Nucleotide sequences of *Pink1* siRNAs, and of the control siRNA.

| Gene | Sequence |
| --- | --- |
| *Net1*-siRNA#1-F | 5’- GCGGCCAUAAACUGUACAUUU-3’ |
| *Net1*-siRNA#1-R | 5’- AAAUGUACAGUUUAUGGCCGC-3’ |
| *Net1*-siRNA#2-F | 5’- CGAAUCUGAGUGUCAGUAUUA-3’ |
| *Net1*-siRNA#2-R | 5’- UAAUACUGACACUCAGAUUCG-3’ |
| *Hace1*-siRNA-F | 5’-GCGGAUGUCAACAUUUGUATT -3’ |
| *Hace1*-siRNA-R | 5’-UACAAAUGUUGACAUCCGCTT -3’ |
| Control-siRNA-F | 5’-UUCUCCGAACGUGUCACGUTT-3’ |
| Control-siRNA-R | 5’-ACGUGACACGUUCGGAGAATT-3’ |


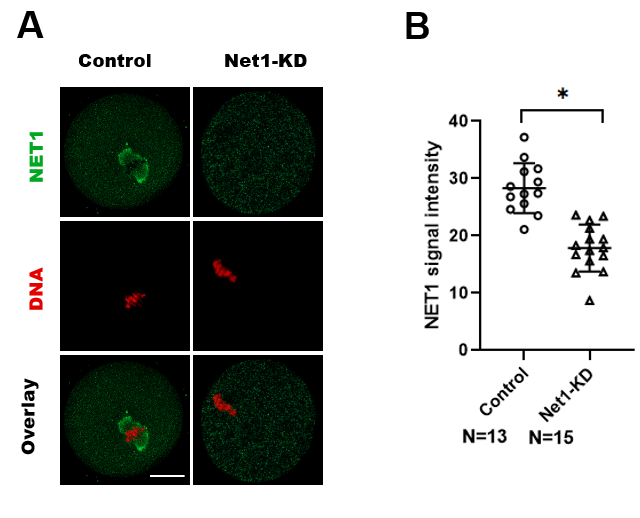


Fig. S1. Effects of siRNA knockdown on NET1 staining in mouse oocytes. (A) Representative confocal figures of control and *Net1*-siRNA injected oocytes stained with anti-NET1 (green) and counterstained with propidiumiodide (red) for nuclear are shown. Scale bar, 20 μm. (B) Quantification of NET1 immunofluorescence shown in A (n=13 for control and n=15 for *Net1*-siRNA group). Results are expressed as mean ± SD from three independent experiments. *, *P* < 0.05
