# Supplementary material for: NET1 is a critical regulator of spindle assembly and actin dynamics in mouse oocytes
Source: Reprod Biol Endocrinol. 2024 Jan 2;22:5. doi: 10.1186/s12958-023-01177-4 (PMC10759572; doi:10.1186/s12958-023-01177-4)
Supplement: Supplementary file 3 — Supplementary Material 3 [file 12958_2023_1177_MOESM3_ESM.docx]

**Original Western blot image of NET1 is a critical regulator for spindle assembly and actin dynamics in mouse oocytes**

Shiwei Wang, Xuan Wu, Mengmeng Zhang, Siyu Chang, Yajun Guo, Shuang Song, Shizhen Dai, Keliang Wu, Shenming Zeng*

State Key Laboratory of Animal Biotech Breeding, National Engineering Laboratory for Animal Breeding, Key Laboratory of Animal Genetics, Breeding and Reproduction of the Ministry of Agriculture, College of Animal Science and Technology, China Agricultural University, Beijing, China

*Corresponding author: [zengsm@cau.edu.cn](mailto:zengsm@cau.edu.cn)

Phone: 18310106460

**This file includes:**

Original Western blot image of Fig. 1A, Fig. 2A, Fig. 5C, Fig. 6B, Fig. 6E, and Fig. 7A.


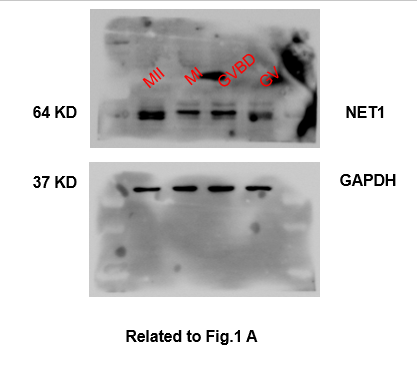


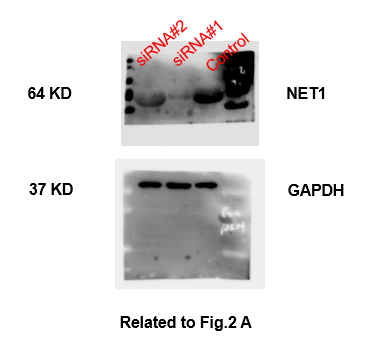


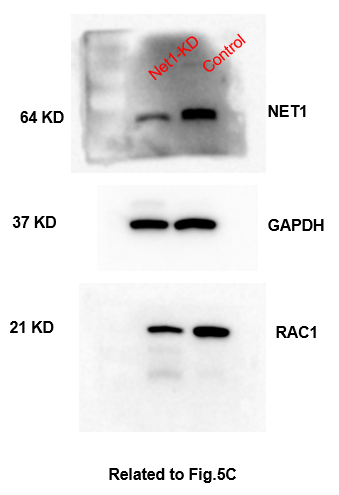


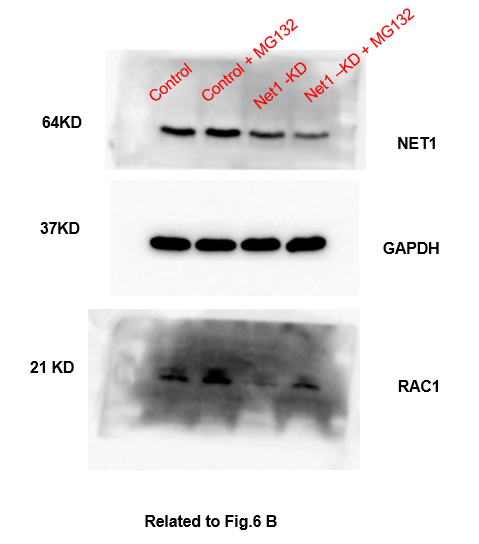


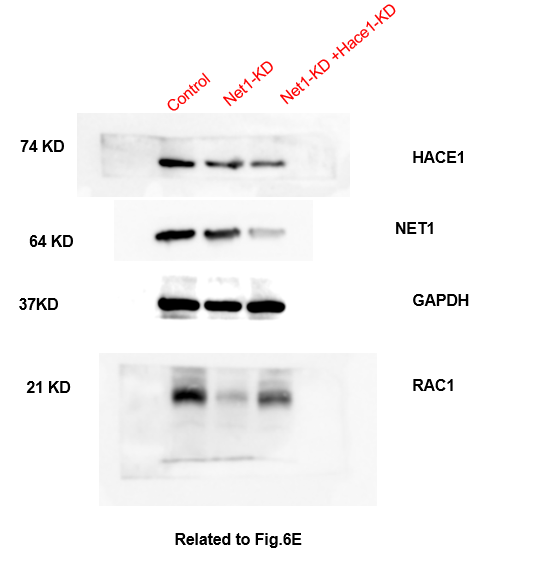


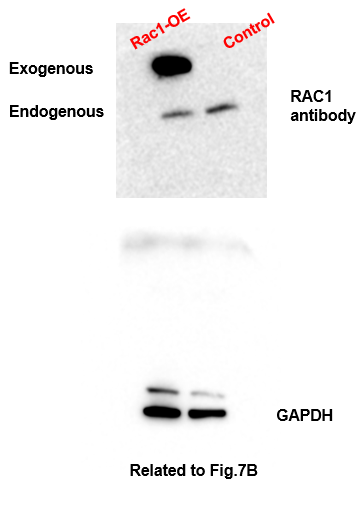


**Antibody specificity test images**

**
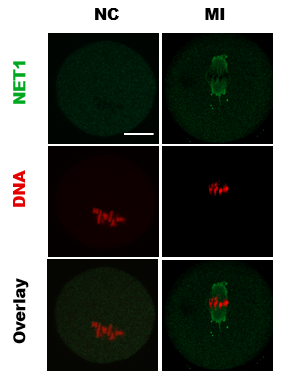

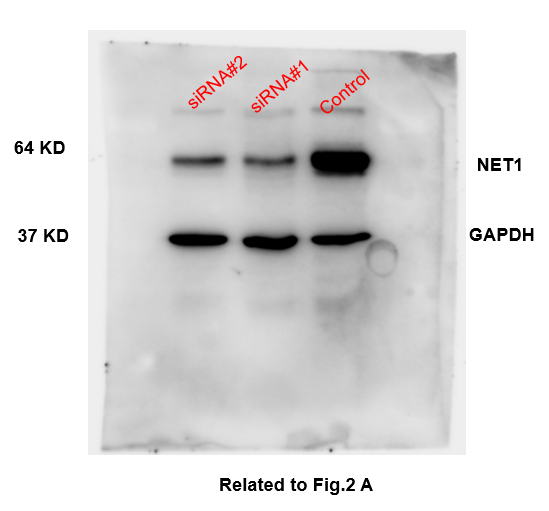

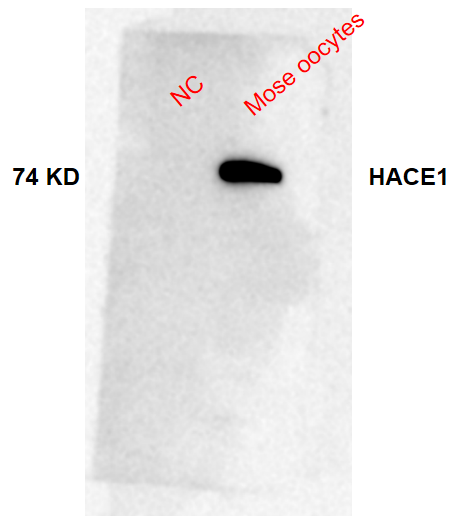

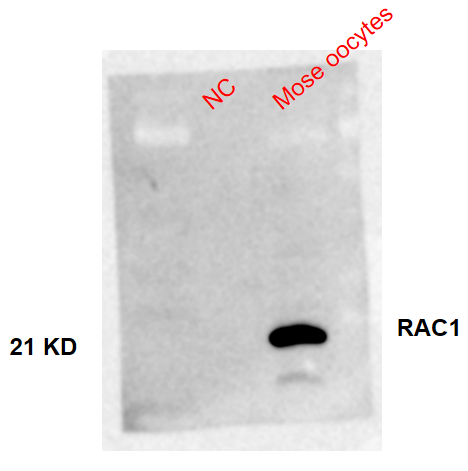

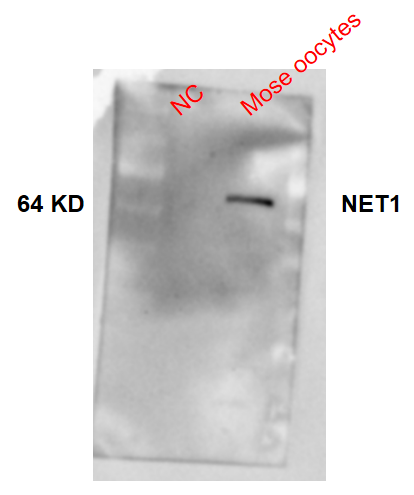
**
